# Cyclosporine A Promotes Bone Remodeling in LPS-Related Inflammation via Inhibiting ROS/ERK Signaling: Studies *In Vivo* and *In Vitro*

**DOI:** 10.1155/2021/8836599

**Published:** 2021-01-07

**Authors:** Yuwei Zhao, Jing Gao, Yarong Zhang, Xueqi Gan, Haiyang Yu

**Affiliations:** State Key Laboratory of Oral Disease, National Clinical Research Center for Oral Diseases, West China Hospital of Stomatology, Sichuan University, Chengdu 610041, China

## Abstract

In some inflammatory diseases of bone, osteogenesis and osteoclasis are uncoupled and the balance is usually tipped resulting in bone destruction. The underlying mechanism of osteogenic dysfunction in inflammation still needs further study. This study is aimed at investigating the effects of cyclosporine A (CsA) on bone remodeling in lipopolysaccharide- (LPS-) related inflammation. *In vivo*, an alveolar bone defect model was established using 10-week-old C57BL/6J mice. The mice were divided into phosphate-buffered saline (PBS), LPS, and LPS+CsA groups. After 3 weeks, micro-CT analysis and histomorphometric evaluation were conducted. *In vitro*, murine osteoblasts were treated with vehicle medium, LPS, LPS+CsA, LPS+extracellular signal-regulated kinase 1/2 (ERK1/2) inhibitor (LPS+PD98059), and LPS+antioxidant (LPS+EUK134). Cell proliferation, osteogenic behaviors, oxidative stress, and ERK signaling were determined. By these approaches, LPS inhibited bone remodeling and promoted oxidative stress accumulation in alveolar bone defects. When animals were treated with CsA, all LPS-induced biochemical changes ameliorated with a marked protective effect. *In vitro*, the reactive oxygen species (ROS) levels in mitochondria increased in LPS-treated osteoblasts, with decreased expression of osteogenic differentiation genes. The CsA, PD98059, and EUK134 presented remarkable protective effects against LPS treatment. CsA effectively enhanced bone remodeling and attenuated oxidative stress caused by LPS via inhibiting ROS/ERK signaling. Taken together, the protective effect of CsA and the inhibitory effect of ERK signaling on the maintenance of mitochondrial function and reduction of ROS levels hold promise as a potential novel therapeutic strategy for inflammatory diseases in bones.

## 1. Introduction

The alveolar bone is a dynamic and continuously changing organ, with osteogenesis and osteoclasis, similar to bone tissues in other parts of the body [1]. This continuous remodeling can prevent and eliminate fatigue-related microinjuries and allow adaptation of bone mass and structure. Some stimulatory factors can cause quantitative changes in the alveolar bone. For example, a physiological inflammatory response can promote the formation of new blood vessels, enhance the function of fibroblasts, and accelerate the process of healing and repair of bone defects [2]. However, an overactive inflammatory response not only inhibits osteoid calcification and new bone formation in the alveolar bone but also promotes osteoclast genesis, resulting in osteolysis and bone resorption [3, 4]. It might be accompanied by inflammatory damage to periodontal tissue destruction in some inflammatory diseases such as osteoporosis, atherosclerosis, diabetes, periodontitis, and chronic apical periodontitis, leading to severe alveolar bone defects that subsequently cause tooth loosening and loss [5, 6]. Controlling inflammation in the alveolar bone might help improve and preserve alveolar ridge height and width.

Cyclosporine A (CsA) was first used to prevent organ rejection after clinical transplantation as a potent immunosuppressant [7]. Later, CsA was regarded as an immunoregulatory and anti-inflammatory drug [8, 9]. Many studies [10, 11] found that CsA inhibited mitochondrial permeability transition pore (mPTP) opening. Enhanced reactive oxygen species (ROS) levels promote the opening of mPTP and reduce the mitochondrial membrane potential (MMP), leading to irreversible mitochondrial dysfunction. In the pathogenesis of some inflammatory diseases, the opening of mPTP induces mitochondria to release a large number of ROS and calcium ions and then cause damage to cell and mitochondrial functions by activating downstream apoptotic signaling pathways [12, 13]. According to recent studies, CsA could protect cells against CoCl_2_-induced hypoxic injury and was a potential therapeutic agent for cardiac hypoxic injury [14], and CsA treatment considerably reduced the H_2_O_2_-induced intracellular generation of ROS [15]. CsA might be developed as a free radical scavenger, but its regulatory mechanism remains unclear. Moreover, the effect of CsA on the healing of bone defect in an inflammatory environment has not been studied.

An increasing number of studies showed that elevated ROS levels could cause oxidative stress-related osteogenesis damage, such as bone remodeling disorders in diabetes, osteoporosis, and periodontitis. Increased levels of oxidative stress impede the proliferation and differentiation of osteoblasts and accelerate cell death. During the pathogenesis of chronic periodontitis, neutrophils produce a large number of ROS in the form of respiratory bursts to kill pathogenic bacteria, while excessive ROS production accelerates cellular senescence, amplifies the inflammatory response, and destroys periodontal tissues [16]. Proinflammatory factors can affect the function of osteoblasts, enhance inflammatory-related bone destruction, and then interfere with the bone remodeling process. Besides, some studies revealed that ROS-mediated oxidative stress was closely related to bone destruction during inflammation [17, 18]. A large number of studies have confirmed that osteoblast apoptosis was related to ROS enhancement. Excessive ROS production caused by external or internal pathways inevitably damaged the lipids and proteins of osteoblasts, leading to fatal cell damage and programmed cell death [19, 20].The normal level of ROS is very important for maintaining bone formation and reconstruction. Whether the ROS is affected and how it is affected during the pathological process of inflammatory diseases need further exploration.

In view of the effect of CsA on antioxidant stress and the close link between ROS and bone remodeling, we hypothesized that CsA promotes the remodeling of alveolar bone defects in the environment of inflammation by suppressing the level of ROS and improving the function of damaged osteoblasts. Using alveolar bone defect model and osteoblasts, we comprehensively evaluated the consequences of changes with CsA treatment on inflammation-specific bone defects. We further delineated the mechanism by which CsA regulates osteoblast function and oxidative stress. Our investigation provides new insight into the role of CsA in inflammatory bone, highlighting the potential the therapeutic application of CsA.

## 2. Materials and Methods

### 2.1. Alveolar Bone Defect Model

A total of 18 male C57BL/6J mice (10 weeks), purchased from the Experimental Animal Center of Sichuan University, were randomly divided into three groups. All mice were reared in the State Key Laboratory of Oral Diseases. The rearing environment (25°C, 55% humidity, and 12 h day and night alternation) was strictly controlled, and water and food were supplied according to the standards. Their weight and health status were all under daily monitoring. The experimental procedures were reviewed and approved by the ethics committee of the State Key Laboratory of Oral Diseases, Sichuan University.

**Figure 1:**
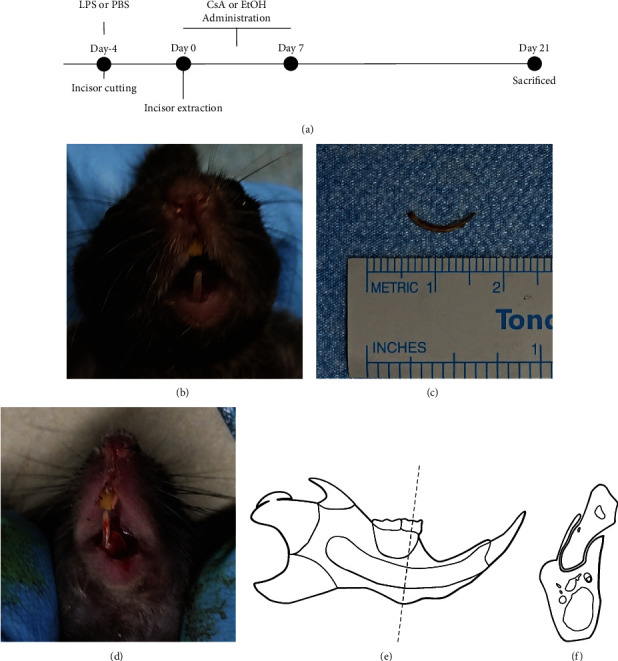
The design of *in vivo* study. (a) Time schedule of *in vivo* study. (b) The crown of the mandibular central incisor was cut. (c) The residual root of the left mandibular incisor was lifted out. (d) The alveolar bone defect model. The dotted line of anatomical diagram of (e) mouse mandible indicates the (f) slices that were performed in the area that was set at the first molar.

The alveolar bone defect model was prepared as described in previous studies [17, 18]. The mice were acclimated in the new rearing environment for at least 7 days. Four days before extraction surgery (Figure 1), the crown of the left mandibular central incisor was cut at the gingival margin under anesthesia and strict disinfection protocol so that the teeth could be smoothly extracted at a later stage. While cutting the crown of the tooth, the central hair on the skin of mice was shaved, and the surgical area was wiped with 10% povidone iodine and deiodinated with 70% ethanol. The mice in the experimental groups received subcutaneous injections of 10 mg/kg LPS (Sigma–Aldrich Corporation, MA, USA) or phosphate-buffered saline (PBS; Solarbio Corporation, Beijing, China) onto the calvaria. Four days after tooth extraction, the residual root of the left mandibular incisor was extracted under anesthesia and strict disinfection protocol. The mice were fed with a sterile soft diet after the extractions for proper wound healing.

The LPS-treated mice were divided into two groups. The residual root was extracted, and the mice received an intraperitoneal injection of 10 mg/(kg · day) CsA (Sigma–Aldrich Corporation) according to the studies by Chen et al. [21] and Li et al. [22] or ethanol solution for seven consecutive days. The three experimental groups were abbreviated as follows: (1) PBS- and EtOH-injected group (PBS), (2) LPS- and EtOH-injected group (LPS), and (3) LPS- and CsA-injected group (LPS+CsA). The mice were sacrificed on day 21 after tooth extraction.

### 2.2. Micro-CT Imaging

The mandible bone free of the skin and the outer layer muscle was harvested 21 days after the extraction and fixed with 4% formalin buffer at 4°C for 2 days. Micro-CT scans were taken using micro-CT 50 (Scanco Medical, Switzerland) with a voxel size of 20 *μ*m and an energy setting of 60 kV and 667 mA. RAW images were reconstructed and analyzed using the Scanco Medical Evaluation and Visualizer software. The region of interest (ROI) was defined to cover the newly formed bone area and the total area in the tooth socket, and a total of 30 successive images were selected from ROI for reconstruction and analysis. The bone volume fraction (BV/TV, %) and bone mineral density (BMD, mg/cc) were measured at all time points.

### 2.3. Hematoxylin–Eosin and Immunohistochemical Staining

The fixed samples were subsequently decalcified for 4 weeks in EDTA–glycerol solution, embedded in paraffin, and sectioned in the coronal plane at a thickness of 4 *μ*m. After deparaffinization, hematoxylin–eosin (HE) staining and immunohistochemical staining were performed. Immunohistochemical staining samples were incubated with anti-alkaline phosphatase (ALP) antibody (Abcam, MA, USA; 1 : 2000), anti-osteocalcin (OCN) antibody (Abcam; 1 : 200), anti-osteoprotegerin (OPG) antibody (Abcam; 1 : 200), anti-RANKL antibody (Abcam; 1 : 200), anti-heme oxygenase-1 (HO-1) antibody (Abcam; 1 : 1000), anti-glutathione peroxidase (GPX) antibody (Abcam; 1 : 50), anti-superoxide dismutase 1 (SOD1) antibody (Abcam; 1 : 500), and anti-monocyte chemotactic protein-1 (MCP-1) antibody (Abcam; 1 : 500). Then, the procedures were performed following standard protocols. Quantitative analysis was performed using the ImageJ software (Rawak Software Inc., Germany).

### 2.4. Cell Culture and Treatment

Murine osteoblasts were generated as described in previous studies with some modifications [23]. In brief, calvarias from newborn mice were dissected aseptically and treated with 0.1% collagenase and 2.5% trypsin. Six populations (I through VI) were obtained after sequential digestion for ~10, 20, 30, 40, 50, and 60 min, respectively. Populations I and II, containing periosteal fibroblastic cells, were discarded. Populations III and IV, containing osteoblast precursors and/or immature osteoblasts, and populations V and VI, containing mature osteoblasts, were cultured in *α*-MEM containing 10% fetal bovine serum (FBS), 2 mM glutamine, and 1% penicillin/streptomycin at a final concentration of 100 IU/mL and 100 *μ*g/mL (Life-Technology, CA, USA). This basic medium was replenished every 3 days. The cells were cultured at 37°C in a humidified atmosphere of 5% CO_2_/95% O_2_.

The drugs were prepared as stock solutions and diluted to working concentrations immediately before use according to manufacturers' protocols. The osteoblast cells were treated with or without LPS (100 ng/mL; Sigma–Aldrich Corporation), CsA (1 *μ*M; Sigma–Aldrich Corporation) according to the study by Yeo et al. [24], ERK1/2 inhibitor PD98059 (10 *μ*M, Sigma–Aldrich Corporation), and EUK134 (10 *μ*M, Sigma–Aldrich Corporation) for 24 h in the basic medium or differentiation medium prior to biochemical and molecular assays.

### 2.5. Cell Viability Assay

The osteoblast cells were plated at 10^4^ cells/well in 96-well plates and cultured under variable conditions as indicated. The cell viability assay was performed using a cell counting kit 8 (CCK-8, Dojindo, Minato-ku, Tokyo, Japan) following the manufacturer's protocol. After LPS stimulation, the cells were transferred to 100 *μ*L of a fresh medium containing 10 *μ*L of reagent mixture and incubated at 37°C for 1.5 h. The plate was gently shaken for 10 s, and the absorbance was measured at 450 nm in a microplate reader (Thermo Scientific, Life Technologies Co., NY, USA).

### 2.6. Cell Differentiation Assay

After drug exposure, the cell monolayer was gently scraped off on the ice and lysed by the ultrasound method. After centrifugation at 10,000 rpm for 5 min at 4°C, the supernatant was used for measuring intracellular ALP activity using an ALP activity assay kit (Jiancheng Bioengineering Institute, Nanjing, China). The absorbance of the reactive volume was detected at 520 nm.

For ALP staining assay, the osteoblast cells were plated at 5 × 10^5^ cells/well in 24-well plates and stimulated with a differentiation medium for 1 week. ALP staining was performed using a standard protocol. Briefly, the cells were gently washed with 1% PBS twice and fixed with 4% paraformaldehyde for 10 min. After incubation with 4% paraformaldehyde (PFA), the cells were gently washed with 1% PBS twice and subjected to ALP staining reagent (Sigma–Aldrich Corporation) following the manufacturer's protocol. The staining results were captured using a digital camera (Canon 60D; Canon, Tokyo, Japan).

Regarding the mineralization assay, the cells were seeded at 10^5^ cells/well in 12-well plates and stimulated with an osteogenic differentiation medium and indicated reagents for 3 weeks. The medium was replenished every 3 days. For detecting osteogenic differentiation, bone nodule formation was examined using Alizarin red staining. Briefly, the cells were fixed with 4% PFA for 30 min and washed with PBS three times. Alizarin red solution 2%, pH 4.1–4.3, was added to the cells for 30 min at 37°C and then washed with PBS.

### 2.7. Mitochondrial Function

The cells were seeded in 24-well plates at a density of 10^5^ cells/well. For mitochondrial ROS determination, the cells were costained with 2.5 *μ*M MitoSOX Red (Molecular Probes) and 150 nM Mitotracker Green (Molecular Probes) for 30 min at 37°C. For determining MMP, the cells were costained with 150 nM tetramethylrhodamine methyl ester (TMRM, Molecular Probes) and 150 nM Mitotracker Green (Molecular Probes) for 30 min at 37°C. After staining, the cells were gently washed with 1× PBS and photographed using an Olympus IX71 fluorescent microscope (400x magnification). The excitation wavelength was 543 nm for MitoSOX and TMRM and 488 nm for Mitotracker Green. The ImageJ software was used for quantification and measurement of fluorescent intensity.

For measuring adenosine triphosphate (ATP) levels, the cells were plated at 10^3^ cells/well in 96-well plates, and the ATP level was detected using an ATP assay kit (Millipore) after drug treatment. Subsequently, the cells were treated with 100 *μ*L of nucleotide-releasing buffer for 5 min at room temperature with gentle shaking, 1 *μ*L of ATP-monitoring enzyme was added into the cell lysate, and the sample was read for 1 min in a microplate reader (Thermo Scientific Varioskan Flash).

The mitochondrial respiration complex activity was measured in osteoblast homogenates as described in previous studies [25, 26]. Briefly, the cells under different culture conditions were harvested, homogenized, and sonicated in the isolation buffer (pH 7.2) containing 225 mM D-mannitol, 75 mM sucrose, 2 mM K_2_HPO_4_, and 5 mM HEPES. Further, 10–50 *μ*g mitochondrial fractions were used for complex activity assay. In a previous report [27], ROS significantly decreased complex III (ubiquinol cytochrome c oxidoreductase) activities in osteoblasts. Therefore, the activity of this respiratory chain component was measured.

### 2.8. Quantitative Real-Time Polymerase Chain Reaction and Western Blot Analysis

Total RNA was extracted with the RNA extraction kits (TaKaRa MiniBEST Universal, Takara Bio Inc., Shiga, Japan), and the concentration and purity of extracted RNA were evaluated using a NanoDrop spectrophotometer (Thermo Scientific, USA). After synthesizing cDNA, polymerase chain reaction (PCR) was performed with a real-time PCR system (Applied Biosystems, USA) using SYBR Premix Ex Taq II (TaKaRa MiniBEST Universal). The primer sequences are listed in Table 1. The expression of detected genes was analyzed using the 2^−*ΔΔ*CT^ method. The Western blot procedures used were described in a previous study [28]. Primary antibodies used included anti-ERK, anti-p-ERK, and anti-*β*-actin (Abcam, Cambridge, MA).

### 2.9. Statistical Analysis

All assays were repeated in three independent experiments. Data were presented as mean ± standard deviation. Significance was determined using the Student *t*-test for pairwise comparison and one-way analysis of variance with the Bonferroni posttest for multiple comparisons using the GraphPad Prism 7.0 software (GraphPad Software, Inc., CA, USA). A *P* value <0.05 indicated a statistically significant difference.

## 3. Results

### 3.1. CsA Attenuated LPS-Induced Osteoblast Dysfunction

**Figure 2:**
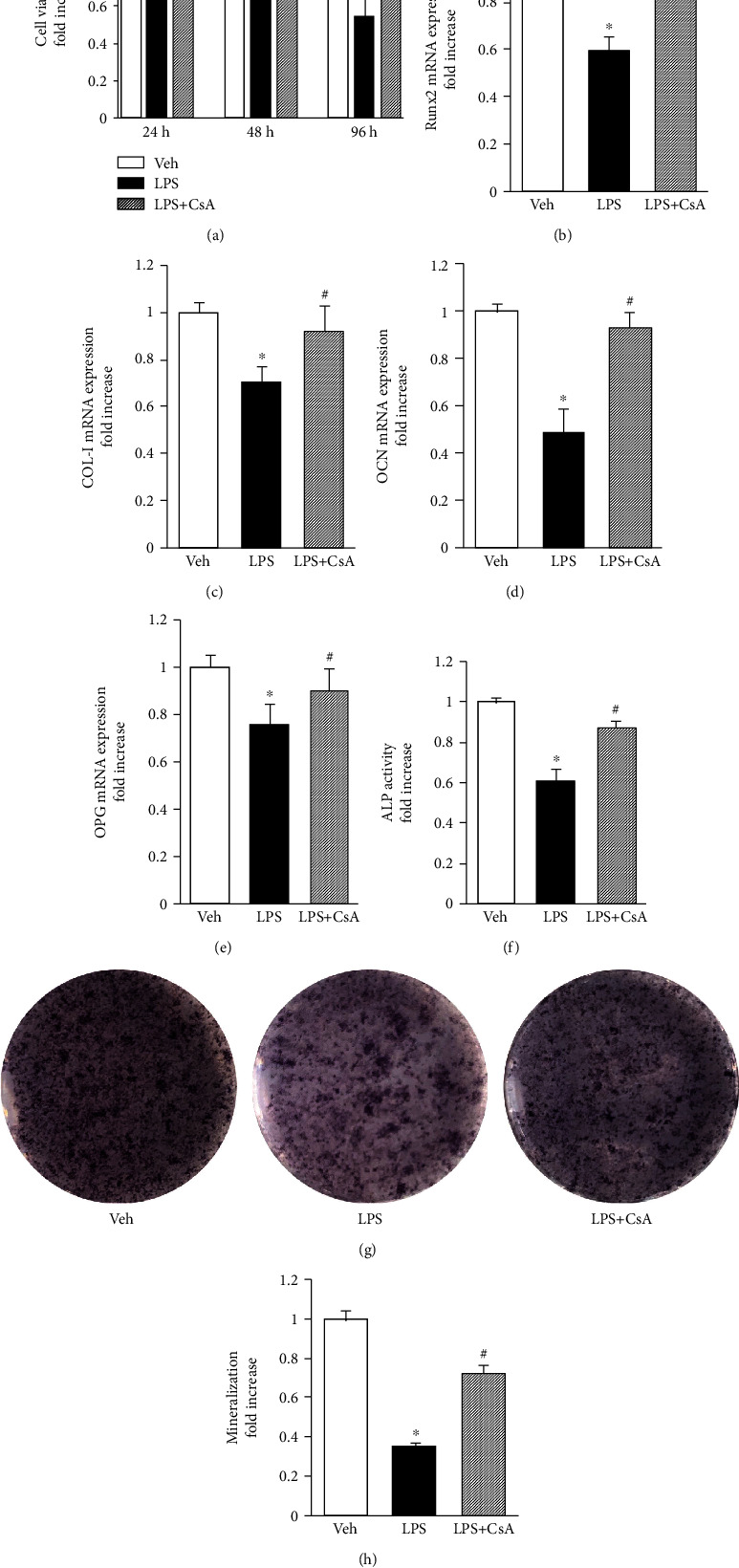
CsA attenuated LPS-induced osteoblasts dysfunction. (a) Cell viability evaluated by the CCK-8 assay at 24, 48, and 96 h, *n* = 6. Relative expression of (b) Runx2, (c) COL-I, (d) OCN, and (e) OPG detected at 24 h by RT-qPCR analysis, *n* = 3. (f) ALP activity was detected by the ALP assay, *n* = 3. (g) ALP staining after an osteogenic induction of 7 days, *n* = 3. (h) Quantification of mineralization nodules in different groups, *n* = 3. ^∗^*P* < 0.05 vs. PBS group; ^#^*P* < 0.05 vs. LPS group.

Excessive inflammation can cause cytotoxicity, interfere with cell growth, and induce cell apoptosis. Cytotoxicity was measured using the CCK-8 assay to determine the toxicity profile. As shown in Figure 2(a), the cell viability did not decrease significantly after treatment with 100 ng/mL LPS for 24 h. Also, CsA treatment displayed a nontoxic effect on cells. The survival of cells pretreated with CsA in the presence of LPS significantly increased compared with the survival of LPS-treated cells for 48 and 96 h (*P* < 0.05)(Figure 2(a)), suggesting that CsA suppressed LPS-induced cytotoxicity.

ALP activity and staining and the expression of calcium deposition- and osteogenic differentiation-related genes were determined to assess the effect of CsA on osteoblast differentiation in an inflammation model. CsA significantly enhanced the expression of osteogenic differentiation-related genes (*Runx2*, *COL-I*, *OCN*, and *OPG*) compared with that in the group treated with LPS alone (Figures 2(b)–2(e)). When osteoblasts were pretreated with CsA in the presence of LPS, the cellular ALP activity, which was one of the major osteoblast differentiation markers, significantly increased. Consistent with ALP activity assay, the results of ALP staining showed that that the expression of ALP-positive cells was significantly less in the presence of LPS than in controls; however, CsA promoted the expression of ALP (Figures 2(f) and 2(g)). Only slight bone nodule formation was observed in the cells exposed to LPS; however, CsA showed a significant recovery effect on LPS-induced mineralization (Figure 2(h)). These data indicated that CsA attenuated LPS-induced osteoblast dysfunction.

### 3.2. CsA Attenuated LPS-Induced Osteoblast Mitochondrial Dysfunction

**Figure 3:**
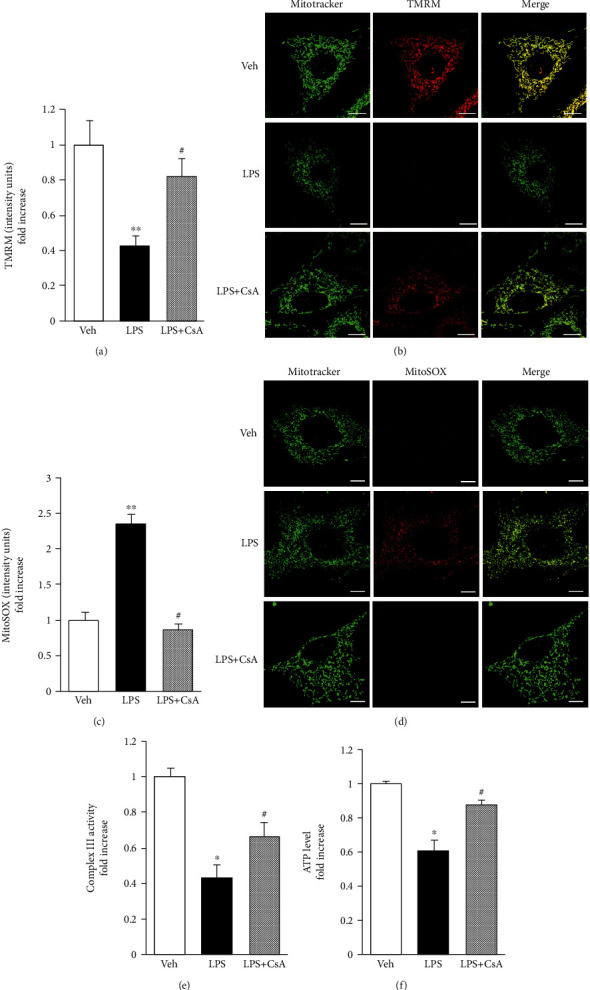
CsA attenuated LPS-induced OB mitochondrial dysfunction. (a) TMRM staining intensity, *n* = 3. (b) Representative images of TMRM staining. (c) MitoSOX staining intensity, *n* = 3. (d) Representative images of MitoSOX staining. (e) Complex III activity was detected by complex activity assay, *n* = 3. (f) ATP production was detected by an ATP assay kit, *n* = 3. Scale bar = 5 *μ*m. ^∗∗^*P* < 0.01 vs. PBS group; ^∗^*P* < 0.05 vs. PBS group; ^#^*P* < 0.05 vs. LPS group.

ROS are natural by-products produced during cell metabolism. However, under some pathological conditions such as inflammation, the production and accumulation of ROS increase significantly and cause serious damage to the cellular structure. ROS can participate in mediating osteoblast dysfunction caused by LPS by regulating related signaling pathways [29, 30]. Increased ROS levels in mitochondria can interfere with the normal mitochondrial functions, such as decreased MMP, inhibited oxidative respiratory chain function, and decreased ATP production [31, 32]. MitoSOX Red, a highly specific dye, was used to identify and label ROS in mitochondria so as to evaluate mitochondrial function more carefully. A specific fluorescent dye TMRM and kits were used to measure inner MMP, ATP levels, and complex III activity. The TMRM staining results showed that the staining intensity decreased in the LPS group compared with the vehicle control group (Figures 3(a) and 3(b)) and returned to normal after CsA treatment. In the vehicle control group, cells showed weak red fluorescence in the mitochondria. However, the red fluorescence intensity in the mitochondria increased significantly in the LPS group compared with the vehicle control group. Also, the red fluorescence intensity in the mitochondria significantly reduced after CsA treatment (Figures 3(c) and 3(d)). Moreover, complex III activity and ATP production followed the same trend. They decreased after LPS treatment and increased to a normal level under the action of CsA (Figures 3(e) and 3(f)). Besides the changes in ROS levels in mitochondria, this study found that LPS could interfere with oxidative metabolism balance in osteoblasts, leading to mitochondrial dysfunction. Moreover, CsA could protect cells from LPS-induced oxidative stress.

### 3.3. CsA Promoted Bone Formation in Alveolar Bone Defects under an LPS Environment

**Figure 4:**
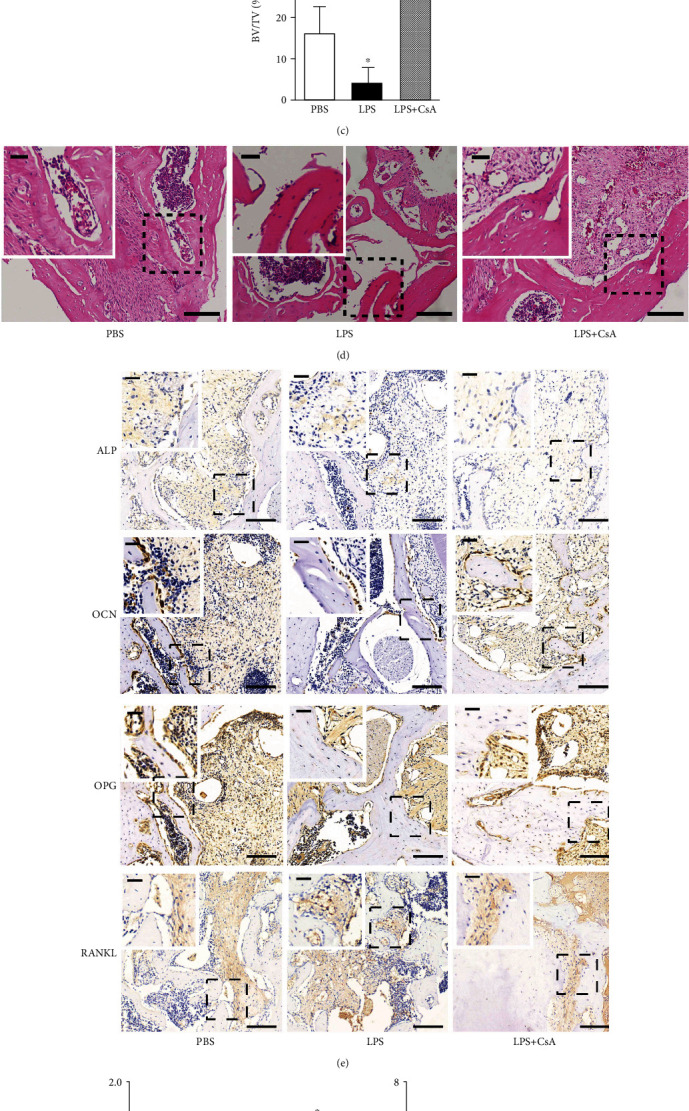
CsA promotes bone formation under an LPS environment *in vivo*. (a) Micro-CT of alveolar bone defect. (b) BMD results, *n* = 6. (c) BV/TV results, *n* = 6. (d) HE staining. (e) Immunohistochemistry staining of ALP, OCN, OPG, and RANKL. (f) IOD values in each group, *n* = 6. (g) Relative expression of ALP, OCN, OPG, and RANKL in the alveolar bone defect area by RT-qPCR analysis, *n* = 6. The longer scale bar = 100 *μ*m, and the shorter scale bar = 20 *μ*m. ^∗∗^*P* < 0.01 vs. PBS group; ^∗^*P* < 0.05 vs. PBS group; ^#^*P* < 0.05 vs. LPS group.

This study is aimed at exploring the effect of CsA on the healing of bone defect areas in an inflammatory environment *in vivo*. Therefore, the morphological examination and detection of the expression of bone formation-related proteins were performed. Micro-CT three-dimensional scanning revealed that the bone formation was significantly better in the LPS+CsA group than in the LPS group (Figure 4(a)). The BMD and BV/TV values significantly increased in the LPS+CsA group than in the LPS group and were even better than those in the PBS group (Figures 4(b) and 4(c)). The HE staining results showed a large amount of tissue fragments and necrotic bone tissue in the LPS-treated group, with no obvious granulation tissue. Granulation-like tissues, similar to those in the PBS group, and a few thinner bones were found in the LPS+CsA group (Figure 4(d)). The aforementioned results indicated that inflammation was not conducive to the bone remodeling process in the alveolar defect. The immunohistochemical staining showed that the staining intensity and mRNA expression levels of runt-related transcription factor 2 (Runx2), ALP, OCN, and OPG were significantly higher in the LPS+CsA group than in the LPS group (Figures 4(e)–4(g)). However, the expression of RANKL was significantly inhibited after cotreatment with CsA (Figures 4(e)–4(g)). The present study supported that inflammation was negative to bone formation in alveolar bone defects, and CsA could promote bone formation in alveolar bone defects.

### 3.4. CsA Effectively Suppressed LPS-Induced Oxidative Stress Status

**Figure 5:**
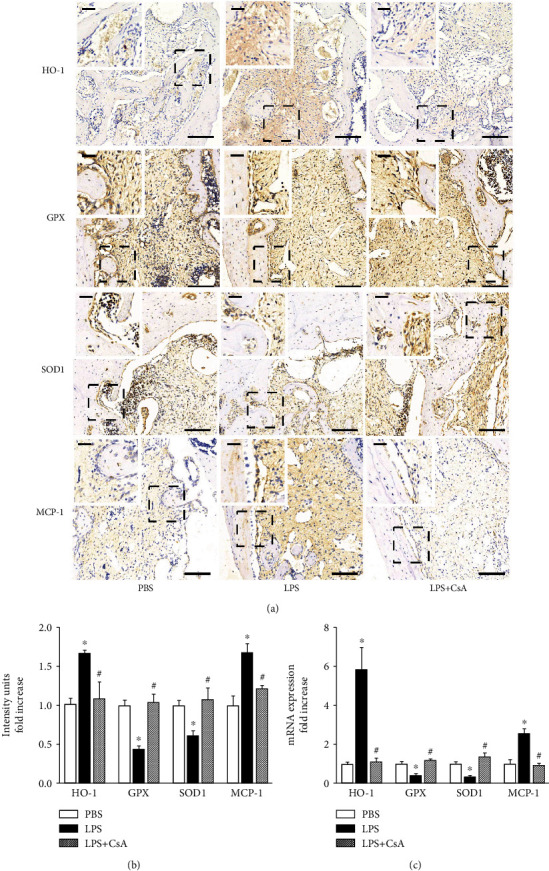
CsA suppressed oxidative stress under an LPS environment *in vivo*. (a) Immunohistochemistry staining of HO-1, GPX, SOD1, and MCP-1. (b) IOD values in each group, *n* = 6. (c) Relative expression of HO-1, GPX, SOD1, and MCP-1 in the alveolar bone defect area by RT-qPCR analysis, *n* = 6. The longer scale bar = 100 *μ*m, and the shorter scale bar = 20 *μ*m. ^∗^*P* < 0.05 vs. PBS group and ^#^*P* < 0.05 vs. LPS group.

This study examined the effect of LPS and CsA on the oxidative stress of alveolar bone defects *in vivo* by determining the expression of oxidative stress-related proteins. A previous study showed that the products produced by HO-1 by catalyzing the degradation of heme caused oxidative stress damage [33]. The activity of HO-1 had a direct impact on the ability to tolerate oxidative stress. SOD1 and GPX constitute a primary defense against oxidative stress; they are involved in regulating mitochondrial ROS [34–36]. The production of ROS is concomitant with oxidative stress, which is exacerbated by the excessive release of cytokines, such as MCP-1, which is an activator of monocytes and can induce oxidative burst [37]. Therefore, the expression of HO-1, SOD1, GPX, and MCP-1 in the alveolar socket was analyzed in this study. Then, whether LPS caused oxidative stress in the alveolar bone defect area and whether CsA alleviated oxidative stress were explored. The immunohistochemical staining results showed that the HO-1 staining intensity was significantly higher in the LPS treatment group than in the PBS treatment group (Figure 5(a)). The expression of HO-1 increased by about 70% in the LPS group compared with the PBS group (Figure 5(b)). After the action of CsA, the staining intensity and the protein and mRNA levels of HO-1 and MCP-1 in the alveolar socket significantly decreased (Figures 5(a)–5(c)). The concentrations of SOD1 and GPX decreased in the LPS group compared with the PBS group (Figure 5(b)). Also, the concentrations and mRNA levels of SOD1 and GPX increased after CsA treatment (Figures 5(b) and 5(c)). LPS increased the level of oxidative stress, which was relieved after CsA treatment.

### 3.5. Antioxidant (EUK134) Reversed LPS-Induced Osteoblast Dysfunction and Mitochondrial Dysfunction

**Figure 6:**
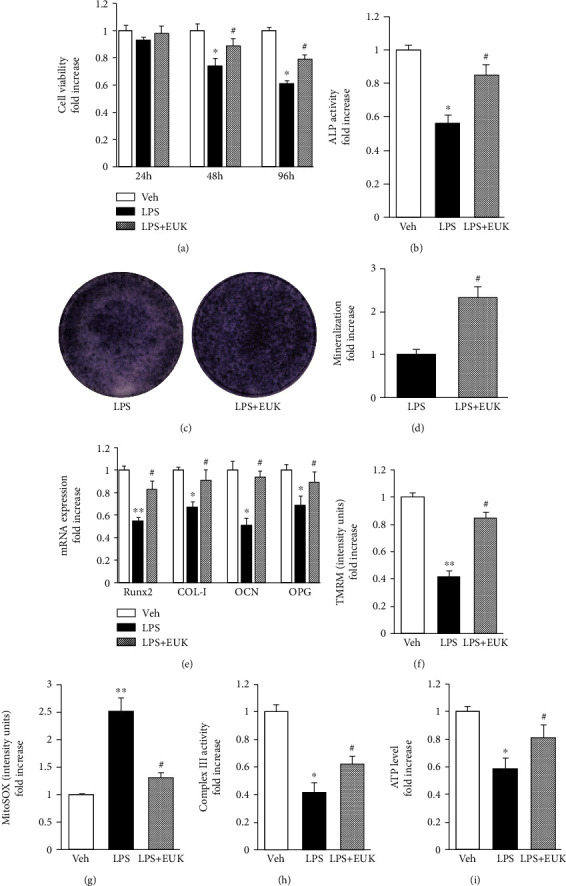
Antioxidant (EUK134) reversed LPS-induced OB dysfunction and mitochondrial dysfunction. (a) Cell viability evaluated by the CCK-8 assay at 24, 48, and 96 h, *n* = 6. (b) ALP activity was detected by the ALP assay, *n* = 3. (c) ALP staining after an osteogenic induction of 7 days, *n* = 3. (d) Quantification of mineralization nodules in different groups, *n* = 3. (e) Relative expression of Runx2, COL-I, OCN, and OPG detected at 24 h by RT-qPCR analysis, *n* = 3. (f) TMRM staining intensity, *n* = 3. (g) MitoSOX staining intensity, *n* = 3. (h) Complex III activity was detected by complex activity assay, *n* = 3. (i) ATP production was detected by an ATP assay kit, *n* = 3. ^∗∗^*P* < 0.01 vs. PBS group; ^∗^*P* < 0.05 vs. PBS group; ^#^*P* < 0.05 vs. LPS group.

Since mitochondrial oxidative stress is important in osteoblast dysfunction, the study tested whether antioxidant treatment could recover the LPS-induced osteoblast dysfunction. EUK134, an antioxidant, is a biomimetic of SOD2 and catalase. As shown in Figure 6, the osteoblasts treated with EUK134 in the presence of LPS significantly inhibited LPS-induced toxic effect on cell viability (Figure 6(a)) and increased cellular ALP activity (Figure 6(b)), ALP expression (Figure 6(c)), and bone nodule formation (Figure 6(d)). Moreover, EUK134 supplementation significantly enhanced the expression of osteogenic differentiation genes (*Runx2*, *COL-I*, *OCN*, and *OPG*) compared with that in the group treated with LPS alone (Figure 6(e)). The results demonstrated that the antioxidative treatment attenuated LPS-induced osteoblast dysfunction.

Considering increased mitochondrial ROS in LPS-treated osteoblasts, LPS was an important contributor of oxidative stress to mitochondrial dysfunction. The study next determined whether antioxidant treatment could rescue the altered mitochondrial function. The results showed that deficits in the membrane potential (Figures 6(f) and 6(g)) and complex III activity (Figure 6(h)) in the osteoblast inflammation model were reversed following treatment with EUK134. Moreover, EUK134 administration suppressed ATP production in LPS-treated osteoblasts (Figure 6(i)). Collectively, the data indicated that EUK134 conferred protective effects on mitochondrial function relevant to LPS-derived mitochondrial toxicity in osteoblasts.

### 3.6. ERK1/2 MAPK Was Involved in LPS-Induced Osteoblast Dysfunction

**Figure 7:**
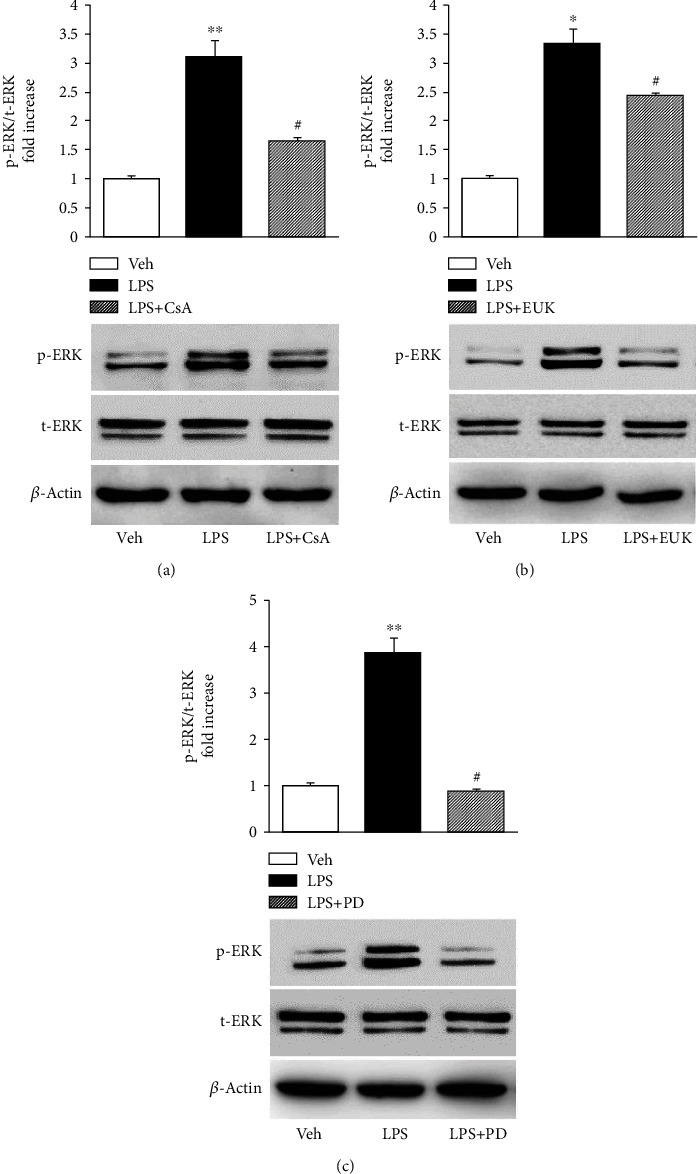
Effect of CsA and ERK1/2–MAPK was involved in the LPS-induced osteoblast dysfunction. (a–c) Ratio of phosphorylated ERK to total ERK levels. p-ERK: phosphorylated ERK (1/2); t-ERK: total ERK (1/2); t-ERK remains mostly unchanged. Representative immunoblots are shown in the lower panel. ^∗∗^*P* < 0.01 vs. PBS group; ^∗^*P* < 0.05 vs. PBS group; ^#^*P* < 0.05 vs. LPS group.

Mitogen-activated protein kinase (MAPK) signaling pathway is known to be involved in both oxidative stress and osteoblast differentiation [38–40]. Moreover, previous studies demonstrated that oxidative stress induced MAPK activation, which was linked to abnormal mitochondrial function [25]. Besides, the inhibition of the MAPK signaling pathway downregulated the expression of inflammatory factors, thereby improving the prognosis of inflammatory diseases [41]. The phosphorylation of MAPK was first analyzed by immunoblotting to determine whether the MAPK signaling pathway was also involved in LPS-induced osteoblast dysfunction. A significantly increased ERK1/2 phosphorylation was observed when osteoblasts were stimulated with LPS. However, the addition of CsA (Figure 7(a)), EUK134 (Figure 7(b)), and PD98059 (ERK pathway inhibitor) (Figure 7(c)) largely abolished ERK1/2 phosphorylation. The total ERK1/2 level did not significantly change under each condition. These results demonstrated that CsA attenuated LPS-induced osteoblast dysfunction through ERK signaling transduction.

### 3.7. ERK1/2 Inhibitor Rescued LPS-Induced Osteoblast Dysfunction and Mitochondrial Dysfunction

**Figure 8:**
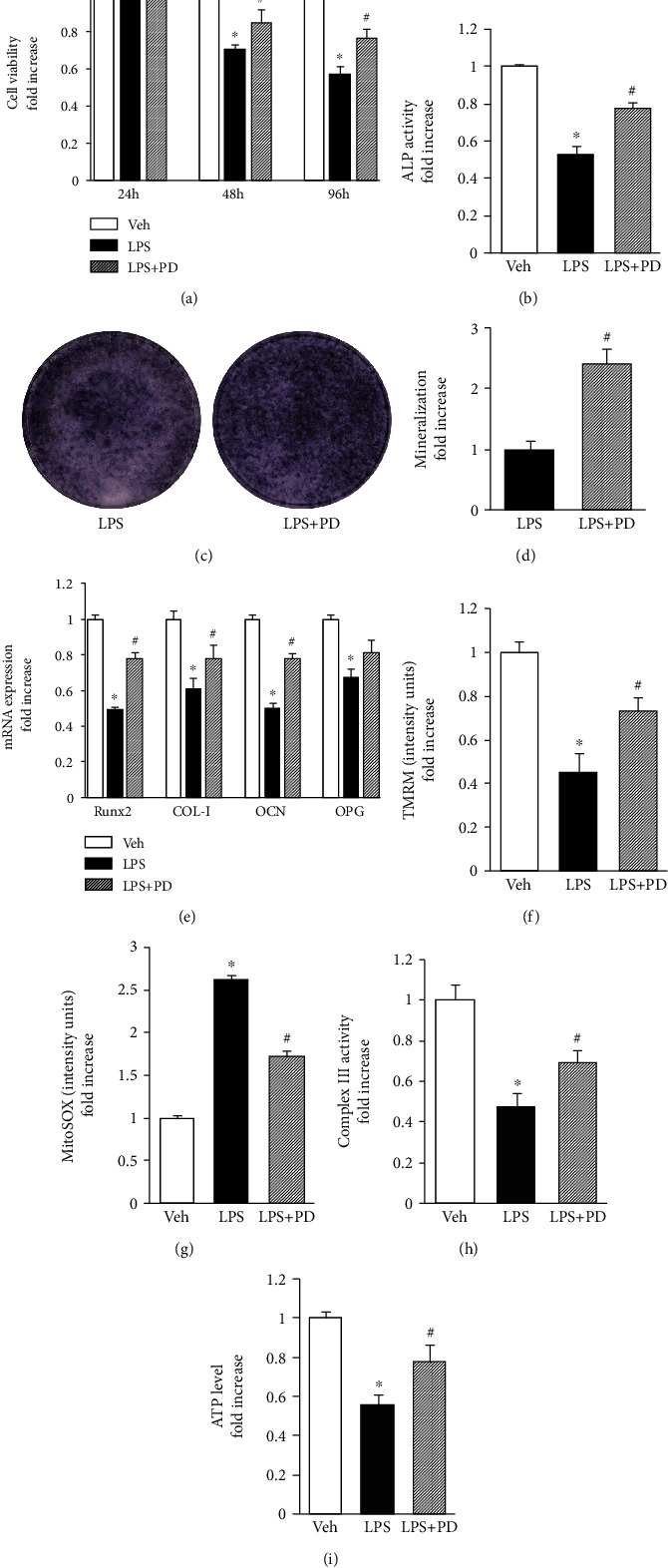
ERK1/2 inhibitor attenuated LPS-induced osteoblast dysfunction and mito dysfunction. (a) Cell viability evaluated by the CCK-8 assay at 24, 48, and 96 h, *n* = 6. (b) ALP activity was detected by the ALP assay, *n* = 3. (c) ALP staining after an osteogenic induction of 7 days, *n* = 3. (d) Quantification of mineralization nodules in different groups, *n* = 3. (e) Relative expression of Runx2, COL-I, OCN, and OPG detected at 24 h by RT-qPCR analysis, *n* = 3. (f) TMRM staining intensity, *n* = 3. (g) MitoSOX staining intensity, *n* = 3. (h) Complex III activity was detected by complex activity assay, *n* = 3. (i) ATP production was detected by an ATP assay kit, *n* = 3. ^∗^*P* < 0.05 vs. PBS group and ^#^*P* < 0.05 vs. LPS group.

The study evaluated whether the ERK1/2 inhibitor could recover LPS-induced osteoblast dysfunction to test the important role of the ERK signaling pathway in the oxidative stress model. PD98059 administration significantly reduced the LPS-induced toxic effect on cell viability (Figure 8(a)). It also enhanced cellular ALP activity (Figure 8(b)), ALP expression, and bone nodule formation (Figures 8(c) and 8(d)). Moreover, EUK134 supplementation significantly enhanced the expression of osteogenic differentiation-related genes (*Runx2*, *COL-I*, *OCN*, and *OPG*) compared with that in the group treated with LPS alone (Figure 8(e)). Then, the study determined whether the ERK1/2 inhibitor could rescue the altered mitochondrial function. The results showed that the deficits in the membrane potential (Figures 8(f) and 8(g)) and complex III activity (Figure 8(h)) in the osteoblast inflammation model reversed following treatment with PD98059. Moreover, PD98059 administration suppressed ATP production in the LPS-treated osteoblasts (Figure 8(i)). These results indicated that the disruption of ERK1/2 expression in osteoblasts might be responsible for abnormal osteoblast and mitochondrial functions induced by LPS.

## 4. Discussion

CsA is a cyclic neutral hydrophobic peptide and a third-generation immunosuppressant. It is often used to treat autoimmune diseases and improve the survival rate of patients after organ transplantation and the survival rate of transplanted organs. Early reports on the role of CsA in bone mineral density were derived from clinical observations, which noted that treatment with CsA and another immunosuppressive drug FK506 was associated with an increased incidence of fractures [42]. However, CsA monotherapy has been reported to significantly increase lumbar vertebral bone density in renal transplant patients [43]. In our study, we studied on the effects of CsA in an alveolar bone defect mouse model under inflammation and investigated whether and how CsA regulates osteogenic function and mitochondrial function during inflammation using murine osteoblasts. We comprehensively evaluated the oxidative stress, mitochondrial function, osteogenic function, and bone formation *in vivo* and *in vitro*.

Bone remodeling mainly comprises osteoblast-mediated bone formation and osteoclast-mediated bone resorption [16]. This process is strictly regulated by a series of related transcription factors, such as Runx2, BMP-2, and RANKL [1]. Runx2 is an important regulator of osteoblast differentiation. Although Runx2-/- skull cells can normally express BMP-2, osteogenic differentiation cannot be completed *in vivo* and *in vitro* [33]. Runx2 overexpression can accelerate the early differentiation of osteoblasts. The results of the present study showed that Runx2 expression was significantly inhibited in the LPS treatment group. After CsA treatment, the expression level of Runx2 increased significantly. Unlike Runx2, RANKL is an important regulatory factor that promotes osteoclast formation and induces osteoclast differentiation. The overexpression of RANKL in osteoblasts can enhance osteoclast activity and promote bone resorption [44]. In the present study, the expression of RANKL in the alveolar bone defects and osteoblasts was significantly upregulated after LPS treatment, while it was downregulated after treatment with CsA and was close to normal physiological levels. Previous study demonstrated that CsA significantly inhibited the dexamethasone-induced decrease in MMP and cell viability reduction in primary cultured osteoblasts [45]. Some studies suggested that CsA might reduce the risk of glucocorticoid-induced osteopenia in patients undergoing transplantation not only by serving as a glucocorticoid-sparing agent but also transiently stimulating bone formation more than bone resorption [43, 46]. The study indicated that LPS correlated with a reduction in cellular ALP activity, a decrease in calcium mineralization, and reduced production of osteogenic genes, which was consistent with previous results showing that the cytotoxicity of LPS resulted in osteoblast dysfunction. In brief, our study showed that LPS treatment suppressed both the expression of early osteogenic markers and ALP activity in murine osteoblasts. We also observed impaired cellular viability in osteoblasts, suggesting that osteogenic activity of osteoblasts was inhibited in an inflammatory environment, while CsA reversed these effects.

*In vivo*, the present study found that the injection of LPS could inhibit bone formation in the mandibular central incisor tooth extraction site. However, no significant inhibition was observed in the LPS and CsA cotreated groups, and surprisingly, it was even better than that in the PBS group. The BMD and BV/TV values of the alveolar bone defects significantly reduced after LPS treatment. However, when the mice were treated with CsA, the osteogenic damage performance in the alveolar bone defects significantly improved. The results of HE staining showed that bone fragments increased, the number of inflammatory cells increased, and no obvious granulation tissue was seen in the LPS-treated group, indicating that LPS had a strong inhibitory effect on bone healing in the bone defect area. After treatment with CsA, granular tissues similar to those in the PBS group were seen in the alveolar bone defects, indicating that CsA might be involved in bone formation disorders caused by LPS. In published papers, CsA was reported to have acute anti-inflammatory effects in immunocompetent animals [47]. Moreover, CsA has become one of the immunosuppressive drugs widely used for the treatment of ocular inflammation [48]. The aforementioned results indicated that the changes in the expression levels of bone formation-related transcription factors in the alveolar bone defects of mice and in osteoblasts were consistent with the results of the morphological examination, revealing the important regulatory effect of CsA on the remodeling process of alveolar bone defects in inflammation as well as on the osteoblast function.

Considering the relationship among CsA, inflammation, and oxidative stress, we focused on the role of ROS. ROS induced oxidative stress is the main cause of cell damage in many inflammatory diseases. Since excessive ROS production may be an important factor in bone defects under inflammation, it will be of great significance to study the molecular characteristics of these effects. Mitochondria not only produce ROS but are also the principal target of ROS attacks. Impaired mitochondrial function can lead to increased ROS generation and result in oxidative injury in the cells and tissues. Komarova et al. suggested that mitochondrial activity and the principal pathways of energy metabolism were vital in the osteoblast function. The respiration, ATP production, and transmembrane potential in mitochondria significantly increased during osteoblast differentiation [49]. All these findings indicated that the maintenance of normal mitochondrial function contributed a lot to the normal functioning of osteoblasts. In the present study, LPS resulted in mPTP opening (TMRM decrease). Also, complex III activity and ATP levels decreased, while these adverse effects on mitochondrial function were reversed by CsA treatment. When the ROS levels exceeded the capacity of the cell in general and the mitochondria in particular to scavenge and render themselves harmless, the resulting oxidative stress initiated mitochondrial permeability transition [50], which then in turn potentiated the oxidative stress. In the present study, LPS was found to increase ROS production in mitochondria, as shown by MitoSOX staining. *In vivo*, results from immunohistochemistry showed that the expression of HO-1, an oxidative stress regulator, was increased. And we observed an increased activator of monocytes (MCP-1) and decreased free radical scavenger (GSH and SOD1) in inflammatory mice. CsA can effectively suppressed oxidative stress status and mitochondrial dysfunction induced by LPS.

Mitochondrial dysfunction is a consequence of oxidative damage caused by increased oxidant levels. Therefore, decreasing oxidant generation and oxidative damage should be an effective way to inhibit mitochondrial impairment [51]. In addition, the present study showed that EUK134 supplementation, an antioxidant drug to suppress mitochondrial ROS generation, not only blunted oxidative stress but also significantly rescued mitochondrial dysfunction against LPS toxicity. Furthermore, the oxidative stress in LPS-induced inhibition of osteoblast differentiation was also recovered by EUK134 administration. All these results further confirmed that LPS-induced oxidative stress was crucial in the osteoblast dysfunction, including mitochondrial function and osteogenic effect. However, the mitochondrial ROS level was significantly lower in cells treated with CsA. These data confirmed that CsA could promote ROS clearance or reduce intracellular ROS formation in the cells, suggesting that CsA treatment protected osteoblast cells from LPS-induced inhibition of osteogenic differentiation.

MAPK signaling pathways are known to be involved in both oxidative stress and osteoblast differentiation. The present study demonstrated that the levels of ERK1/2 phosphorylation significantly increased in LPS-treated osteoblasts. In agreement with the present findings, other studies showed that LPS suppressed osteoblastic differentiation of bone marrow progenitors and promoted osteoblast apoptosis or inflammatory osteolysis in an ERK-dependent manner *in vitro* and *in vivo* [52–54]. Moreover, antioxidant EUK134 blocked LPS-induced ERK1/2 activation, indicating a role of LPS-induced oxidative stress in the disruption of signal transduction such as ERK1/2 MAP kinase. Notably, LPS-induced ERK1/2 phosphorylation was blunted in osteoblasts treated with CsA. The addition of the specific ERK1/2 inhibitor (PD98059) resulted in pronounced preservation of mitochondrial function even in the face of LPS insults. Moreover, PD98059 even recovered cellular ALP activity, mineralization, and expression of osteogenic differentiation-related genes inhibited by LPS, suggesting that ERK1/2 was required for oxidative stress-induced inhibition of osteoblastic differentiation in osteoblasts, which was completely coincident with previous findings [38]. All these results indicated the involvement of ROS/LPS-associated ERK1/2 MAPK signaling in the disruption of osteoblast dysfunction. The findings suggested that ERK1/2 was a downstream target of LPS. Thus, it was proposed that LPS-induced inflammation and impaired ROS production/accumulation in mitochondria are responsible for ERK1/2-MAPK activation, leading to osteoblast dysfunction.

## 5. Conclusions

**Figure 9:**
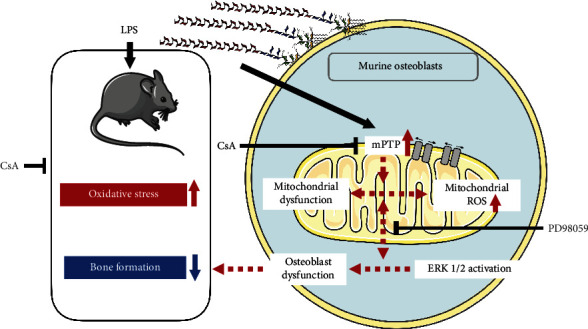
Diagram of CsA mechanism of action on bone remodeling in LPS-related inflammation.

In summary, the data provided new insights into the mechanism of inflammation-induced osteoblast dysfunction in the pathogenesis of inflammatory diseases in bones, especially the role of CsA in this model of alveolar bone defect inflammatory cell model. Oxidative stress is an important cause of reduced bone formation in inflammatory bone defects and mitochondrial dysfunction. CsA can effectively enhance bone formation in alveolar bone defects. Oxidative stress caused by inflammation promoted the opening of mPTP and consequently enhanced ROS production/accumulation, thus activating the ERK signal transduction pathway. All these events disrupt the cell viability and osteogenic effect of osteoblasts, ultimately causing cell injury and dysfunction (Figure 9).

## Figures and Tables

**Table 1:** Primer sequences for real-time qPCR analysis of the mRNA expression.

Genes	Primers (5′–3′)	
Runx2	F: CCCAGCCACCTTTACCTACA	R: TATGGAGTGCTGCTGGTCTG
ALP	F: CCAACTCTTTTGTGCCAGAGA	R: GGCTACATTGGTGTTGAGCTTTT
OPG	F: TTACCTGGAGATCGAATTCTGCTTG	R: GTGCTTTCGATGAAGTCTCAGCTG
RANKL	F: GCAGCATCGCTCTGTTCCTGTA	R: CCTGCAGGAGTCAGGTAGTGTGTC
OCN	F: GGAGGGCAATAAGGTAGTGAACAG	R: ATAGCTCGTCACAAGCAGGGT
COL-I	F: TGACTGGAAGAGCGGAGAGTA	R: GACGGCTGAGTAGGGAACAC
HO-1	F: GCTGGTGATGGCTTCCTTGTA	R: ACCTCGTGGAGACGCTTTACAT
SOD1	F: ATGTGACTGCTGGAAAGGACG	R: CGCAATCCCAATCACTCCAC
GPX	F: CCAGGAGAATGGCAAGAATGA	R: GGAAGGTAAAGAGCGGGTGA
MCP-1	F:GCAGGTCCCTGTCATGCTTCT	R:TGTCTGGACCCATTCCTTCTTG
GAPDH	F: ACTTTGTCAAGCTCATTTCC	R: TGCAGCGAACTTTATTGATG

F: forward; R: reverse.

## Data Availability

The data used to support the findings of this study are available from the corresponding author upon request.
